# Treatment Expenditure Pattern of Epileptic Patients: A Study from a Tertiary Care Hospital, Kolkata, India

**DOI:** 10.1155/2014/869572

**Published:** 2014-02-24

**Authors:** Abhik Sinha, Dulal K. Bhaumik

**Affiliations:** ^1^Department of Community Medicine, R.G Kar Medical College, 1 Khudiram Bose Sarani, Kolkata 700004, India; ^2^Biostatistical Research Center, Biostatistics, Psychiatry and Bioengineering, University of Illinois at Chicago, 1601 W Taylor Street, MC 912, Chicago, IL 60612, USA

## Abstract

*Introduction*. Neurological diseases are very important causes of prolonged morbidity and disability leading to profound financial loss. Epilepsy is one of the most important neurological disorders. It being a cost intensive disorder poses a significant economic burden to the country. *Aims and Objectives*. The study was conducted among the persons with epilepsy (PWE) to assess their expenditure pattern for epilepsy treatment and its rural urban difference. *Materials and Methods*. 315 PWE selected by systematic random sampling and their caregivers were interviewed with the predesigned, pretested semistructured proforma. Subsequently data were compiled and analyzed using SPSS 18.0 software. *Results and Conclusion*. Majority of the study population were in the age group of 16–30 years. Majority belonged to classes IV and V of Prasad socioeconomic status scale. Average total expenditure per month for treatment of epilepsy was 219 INR, mainly contributed by drugs, travel, investigations, and so forth. Rural population was having higher treatment expenditure for epilepsy specially for travel and food and lodging in order to get epilepsy treatment. Wage loss in the last three months was present in 42.86% study subjects which was both affected by seizure episodes and travel for visits. Better district care would have helped in this situation.

## 1. Introduction

In the developing countries like India the focus of health services for many decades has been on prevention, management, and control of communicable diseases. Noncommunicable diseases (cardiovascular, cancer, neurological, mental health disorders, etc.) have recently started to draw equal attention both in health care delivery and for funding clinical researches.

Neurological diseases are important causes of morbidity and disability leading to great financial loss. Epilepsy is one of the most important neurological disorders. It poses a substantial social and economic burden to the country [1].

Good education is an essential factor in acquiring gainful employment. The epileptic children often lag in education as they find it hard to get jobs. Employers do not want to employ a person with epilepsy: when employed and if prone to uncontrolled attacks the person with epilepsy faces a worsened situation. They are not given normal jobs but placed in sultry peripheral low-income assignments. Often these jobs are terminated.

Population based neuroepidemiologic studies in different regions of India have shown that epilepsy constitutes nearly a third to a fifth of all neurological disorders. It has been estimated that India with 6–10 million PWE accounts for nearly 1/5th of the global burden of epilepsy [2]. Study conducted by Sridharan and Murthy [3] had shown prevalence as 5.59 per 1000 with no statistical difference between men and women in urban and rural areas.

Epilepsy is a cost intensive disorder. Developing countries carry 90% of the financial burden of epilepsy as 85% of world's 40 million PWE live in developing countries. Principles of health economics have been introduced to the management of epilepsy in the recent past. Health is an important economic resource and ill health leads to economic burden. Traditionally cost of illness is estimated under direct cost (cost of medical treatment, other nonmedical expenditure such as travel to hospital, etc.), indirect cost (due to lost productivity), and intangible cost (related to the emotional and social impact of illness on the economy). The economic burden due to a disease depends on the age of onset, natural history, impact on quality of life, cost of treatment, rehabilitation, risk of mortality, and several other factors. With the advent of new Antiepileptic Drugs (AEDs) and implementation of surgical programs, the direct cost of epilepsy has increased by many folds. Accordingly the out of pocket expenditure for patients has increased in India and many other developing countries that do not have insurance or social security programs for epilepsy. Increase in the cost of epilepsy care would be an additional strain on the already weak economics in the developing countries. Nevertheless, the potential savings in indirect cost (increased productivity) and intangible costs (improved quality of life) are likely to outweigh the investment in direct cost. According to estimates by International League against Epilepsy (ILAE) and World Health Organization (WHO) nearly 80% of people with active epilepsy living in developing countries are not receiving any treatment. The indirect costs of epilepsy due to disease and its complications are likely to be very high in developing countries due to the large treatment gap [4].

Efforts should be made to reduce the treatment gap in epilepsy. According to Iyer et al. a substantial proportion of the current large treatment gap in epilepsy in developing countries could be minimized by educating the primary care physicians about the diagnosis of epileptic seizures, cost-effective AED treatment, and need-based referral for specialized care [5].

In a landmark multicentric study to study the cost of epilepsy in India the total annual cost per patient amounted to INR 13,755 (USD 344). The total economic burden of epilepsy in India was found to be INR 68.75 billion (1.72 billion USD) which constituted 0.5% GNP of India [4].

In this present scenario where epilepsy has already been declared a public health problem by World Health Organization (WHO) and International League Against Epilepsy (ILAE) this study was planned in the Department of Neuromedicine, Medical College, Kolkata, which is the oldest medical college and a premier hospital in India and caters a huge number of patients from all over the state of West Bengal and some neighboring states regularly.

## 2. Aims and Objectives

The study was conducted among the PWE to assess their expenditure pattern for epilepsy treatment and its rural urban difference.

## 3. Materials and Methods

The present study was an observational, descriptive, cross sectional, and hospital based study. Study area was the Out Patient Department (OPD) of Department of Neuromedicine of Medical College, Kolkata, situated in Kolkata which is a well-known metro city of India. Study period was from May 2009 to April 2010. Study population was the diagnosed epilepsy patients attending the said OPD.

From the last five years' hospital records, the average yearly attendance in the Neuromedicine OPD of Medical College was calculated to be 840 with negligible yearly variation. As the data collection period was decided to be 9 months out of total 12 months of study period, the expected attendance in the period of nine months came to be 630. Considering feasibility, sample size was determined to be 50% of the expected study population that is 315 which was decided to be the study population. The study sample was selected by systematic random sampling technique. Entry of study subject was continued up to the total desired sample size of 315 was arrived.

Inclusion criteria of the study sample were age of the epilepsy patient ≥12 years, willingness to participate in the study, and presence of informant/spouse/parent/near relative with the patient. There was none who refused to participate in the study.

Considering the type of drugs used for treatment of epilepsy the conventional AED included phenobarbitone, phenytoin, carbamazepine, and so forth and newer AEDs included the drugs other than conventional AEDs eg Lamotrigine, Levetiracitam.

Ethical clearance from the concerned authority of Medical College, Kolkata, and informed consent of patients were taken before the study. Study techniques included interviewing the patients and attending caregiver with a predesigned, pretested semistructured proforma and analysis of available patient records. Expenditure for treatment was determined for the last three months by interviewing the patient and his/her caregiver. The mean expenditure per month was calculated from this which was used in the analysis. Socioeconomic class assessment was done by the B.G. Prasad scale for socioeconomic status.

The operational definition of response used in the study was >50% reduction in seizure frequency after 12 weeks of treatment with AED as suggested by Engel et al. [6]. Patients fulfilling the above criteria were categorized as “responders” and the others were regarded as “nonresponders.”

Analysis was doneusing SPSS 18.0 software.

## 4. Results

This cross-sectional study done on 315 epilepsy patients revealed the following results.

Majority of the study population belonged to the age group of 16–30 years (54.6%) were males (66.03%) and were Hindu (69.2%). Mean age of the study population was 29.98 ± 12.70 years. 75.2% were from urban areas, whereas 24.8% were from rural areas. 32.4% of the study population had completed primary education and 14.3% were illiterate. Majority of the study population were unskilled labour (30.2%). 47.6% of the study population belonged to class IV of Prasad scale. 55.2% of the study subjects were on monotherapy. 80% of the patients were on conventional AED and 7.6% were on newer antiepileptic drugs (Table 1).

Mean per capita income for total study population was 678.70 ± 915.8 INR, for males 737.16 ± 1066.98 INR and for females 565.07 ± 491.45 INR and the difference between males and females was not statistically significant (*P* = 0.377). Considering the mean per capita income for rural and urban population, the difference was also found significant (*P* = 0.001).

Expenditure for treatment of epilepsy consisted of the expenditure for drugs, expenditure for travel to Neuromedicine OPD of Medical College, Kolkata, expenditure for food and lodging while coming to visit Neuromedicine OPD of Medical College, Kolkata, and expenditure for investigations. Expenditure for treatment was calculated interviewing the patient for expenditure in these areas in the last three months. Three-month recall has been used to assess the expenditure pattern for epilepsy in the epilepsy under study to reduce recall bias. The mean of the expenditure for three months was used in the analysis as mean monthly treatment expenditure in all the cases.

69.5% of the epilepsy patients received drugs free. The range of expenditure for drug varied from Nil to 672 INR per month in the patients under study (Table 2).

Travel cost to Medical College for 10.1% urban population was nil while for the rural population the minimum cost was 26 INR. The mean travel expenditure of the rural population was 66.47 ± 31.25 INR while that of the urban population was 29.36 ± 18.36 INR and this difference was statistically significant (*U* = 1769.5, *P* = 0.001) (Table 3).

For food and lodging, majority (53%) of the study population had the expenditure of less than 25 INR per month. 43.9% of urban population had no expenditure for food and lodging compared to only 2.6% of rural population. The mean expenditure in this regard was 13.25 ± 13.22 INR, which for the rural population was 26.28 ± 14.04 INR and for the urban population 8.97 ± 9.68 INR. While comparing the rural urban difference, the difference was found to be statistically significant (*U* = 2515.0, *P* = 0.001) (Table 4).

Combining the drug, travel, food, and lodging expenditure along with the expenditure for investigations for epilepsy, the total expenditure per month for treatment of epilepsy was calculated. It was higher for the rural population 306.67 ± 340.59 INR while for the urban population it was 191.28 ± 229.16 INR. But the difference was not statistically significant (*U* = 3575.50, *P* = 0.212) (Table 5).

PWE receiving polytherapy were having higher treatment expenditure for epilepsy than those receiving monotherapy (*U* = 9414.5, *P* < 0.001) (Table 6).

PWE who responded to treatment were having lower treatment expenditure than those who have not responded to treatment (*U* = 1450, *P* < 0.001) (Table 7).

Due to epilepsy, 83.8% of the study population had workdays lost ≤15 days in the last 3 months. The mean workdays lost in the last 3 months by the study population were 11.88 ± 26.26 days and comparing the male female difference, the difference was not observed to be statistically significant.

42.86% of the study population had wage loss. There was no statistically significant difference between rural and urban residence (*Z* = 1.60, *P* = 0.10) so far as presence of wage loss was concerned (Table 8).

## 5. Discussion

Epilepsy constitutes a major public health problem all over the world and also in a developing country like India. Of the 50 million people with epilepsy worldwide, around 80% reside in resource-poor countries, which are ill-equipped to tackle the enormous medical, social and economic challenges posed by epilepsy. The capability to identify people with epilepsy and provide cost-effective care is compromised by widespread poverty, illiteracy, inefficient and unevenly distributed health-care systems, and social stigma and misconceptions surrounding the disease. Several studies have reported that a large proportion of patients with epilepsy in resource-poor countries never receive appropriate treatment for their condition, and many, although diagnosed and initiated on treatment, soon discontinue treatment. The high cost of treatment, a lack of availability of antiepileptic drugs, and superstitious and cultural beliefs contribute to a large epilepsy treatment gap [7].

In the present study mean age of the study population was found to be 29.98 ± 12.70 years. Mean age of the epilepsy patients was found to be 26.24 ± 8.45 years by Basu et al. [8] and 31.2 ± 10.7 years by Thomas et al. [9]. In the present study 75.2% of the study population were from the urban areas which was similar to the observation of Basu et al. [8] and Thomas et al. [9]. In this study 14.3% patients were illiterate. Similar to it, Thomas et al. [9] had observed 11% as illiterate in their study.

The mean per capita per month income was seen as 678.70 ± 915.80, whereas Thomas et al. [4] had seen the total family income per month as 3538 ± 5139 in their multicentric study. In the present study 55.2% were on monotherapy. These findings were almost similar to the findings of Thomas et al. [9] where 74.1% patients were put on monotherapy. In the present study 80% of the study population were having conventional AED, 7.6% were having newer AED, and 12.4% were taking both types of AEDs. The findings were similar to those of Thomas et al. [9] who found them as 71.2%, 13.3%, and 12.5%, respectively.

It has been observed in the present study that 69.5% of the study population obtained drug free of cost and that were mainly from government hospitals The expenditure for drugs for the last 1 year was seen as INR 1224.72 ± 2192.88 (mean expenditure per month was INR 102.06 ± 182.74) in the present study, whereas it was seen as 2149.80 INR and 2276 INR yearly by Thomas et al. [4, 9] in their two studies. The difference might be due to availability of the free medicine in the present study.

In the study by Haroon et al. the monthly costs of each AED prescribed were compared and it was found that much higher cost for some of the newer AEDs like the lamotrigine, levetiracetam than the conventional like sodium valproate, carbamazepine, and so forth [10]. In the present study the PWE receiving polytherapy were having higher treatment expenditure for epilepsy than those receiving monotherapy (*U* = 9414.5, *P* < 0.001).

While considering the expenditure for travel for the study population to visit Medical College in the last year, it was seen as 462.6 ± 328.68 INR (mean expenditure per month as Rs. 38.55 ± 27.39). Thomas et al. [4] found it as 658.50 INR. In rural India Pal et al. [11] observed the total cost as 369 INR for hospital, 350 INR for private doctor, and 120 INR for quack and allopathic practitioners. Here the total out of pocket expenditure was seen to be 2638.2 INR, whereas Thomas et al. [4] have seen it as 3724.8 INR.

42.85% had lost their wages besides direct expenditure in the present study. Thomas et al. [4] had shown the wage lost in monitory terms and it was 1317 INR per year. Mean workdays loss was seen as 11.88 ± 26.26 days in the present study, whereas the mean loss of workdays was about 58 days in study by Thomas et al. The indirect cost related to loss of work may be to the tune of Rs. 6000/—[4]. Study conducted by Thomas et al. had also revealed that most of the epilepsy patients had to travel 70 km or more for medical consultations although a general practitioner was available within 3.4 km from their place of residence. The travel expenses (INR 659) and loss of productivity (INR 1317) due to their long trips together accounted for 14% of total annual cost and were nearly as much as the cost of AEDs [4].

A systemic review was done on 22 studies worldwide on costs of epilepsy of which four were from US, eleven from Europe, two from India, and one each from Hong Kong, Oman, Burundi, Chile, and Mexico. It was found that the studies utilized different frameworks to evaluate costs. However only 12 studies (55%) evaluated direct as well as indirect costs. The range for the mean annual direct cost varied between 40 International Dollar purchasing favour parities (PPS-$) and PPP-$ 4748 (adjusted to 2006 values). Recent studies also observed that AEDs were becoming the main contributor to direct cost. The mean indirect costs ranged between 12 and 15% of the total annual costs [12]. The present study also revealed AED to be a major contributor to total cost so PWE with polytherapy were having higher out of pocket expenditure than monotherapy for epilepsy treatment.

Pillas in another systematic review of 31 epilepsy costs studies found Carbamazepine to be most economic and lamotrigine to be most costly among the AEDs. Cost effectiveness analysis studies were found to be much more credible than cost-benefit analysis. Cost-effective analysis found topiramate to be more cost-effective than lamotrigine and surgical lobectomy to be a very cost-effective treatment in long run [13]. The present study shows that response to treatment is a determinant of treatment expenditure. Nonresponse leads to higher treatment expenditure.

The first comprehensive study on cost of epilepsy in USA was carried out in 1975 [14]. That study estimated the national cost of epilepsy at $3.6 billion for 2.1 million cases. On a per patient basis, the 1975 figures represent US $7440 in 1995, $1150 (15%) for direct treatment-related costs, and $6290 (85%) for indirect employment-related costs [14].

An exhaustive cost of illness study on epilepsy was carried out in UK [15]. This was based on data from National Epilepsy Society and National General Practice Study Group for Epilepsy. A longitudinal cost *profile of epilepsy was calculated, with an average initial direct cost of *£* 611 (US $917) per patient* per annum which decreased after eight years of followup to *£* 169 (US $254) per patient per annum. The cost of newly diagnosed epilepsy in the first year of diagnosis in the UK was *£* 18 million (US $27 million). The total annual cost of established epilepsy in the UK was estimated to be *£* 1930 million (US $2895 million), over 69% of which was due to indirect costs (unemployment and excess mortality). The cost of active epilepsy per patient was approximately *£* 4167 (USA $6251), and of inactive epilepsy *£* 1630 (US $2445) per patient per annum [15].

Thus it can be said from the above discussions that cost of epilepsy care has escalated many folds in the recent past. The high cost of newer antiepileptic drugs, cost of elaborate presurgical evaluation and surgery, account for a large component of direct medical cost. Indirect cost to the society, through lost productivity or premature death, is many times more than the direct cost. The newer drugs have an advantage over the conventional drugs in terms of tolerability, safety, and ease of administration. The benefits in terms of better control of seizures and improvement in quality of life offered by these newer strategies in treatment of epilepsy need to be considered along with the increase in cost. Careful economic evaluation is essential to assess ultimate utility of these interventions in the management of epilepsy at large. Unfortunately, there is little data on this aspect for the physician to apply in his practice [16].

## 6. Conclusion and Recommendation

Thus the study highlights the need of availability of doctors of modern medicine and AED in rural areas, especially in primary health care setting. Efforts should be taken for proper employment of the epilepsy patients. Thus a district care model for epilepsy can be recommended, where training of specialists of medicine and pediatrics of the district regarding epilepsy can be organized by a nodal neurologist from tertiary setting who will in turn provide training to the Block Primary Health Center and Primary Health Center Medical Officers. This referral chain if established can allow proper followup and referral. Primary Health Center Medical Officers in turn can train the peripheral health workers regarding case detection of epilepsy and thus reducing greatly the treatment delay and treatment expenditure. The rural population who are financially weaker have more out of pocket expenditure in treatment of epilepsy. The district care model can help much to lessen the expenditure for travel, food, and lodging and hence the total treatment expenditure for epilepsy. Thus the financial burden on the rural epileptic patients can be greatly reduced. Novel techniques like telemedicine can be implemented as cost-effective measure. Option of surgical treatment of epilepsy may also be considered.

## Figures and Tables

**Table 1:** Baseline information of the study population (*n* = 315).

Variables	Group 1 No. (%)	Group 2 No. (%)	Group 3 No. (%)	Group 4 No. (%)	Group 5 No. (%)	Group 6 No. (%)
Age (in years)	≤15	16–30	31–45	46–60	≥60	
	20 (6.3)	172 (54.6)	72 (22.9)	43 (13.7)	2 (0.6)	

Marital status	Never married	Currently married	Widow/widower	Divorced/separated		
	204 (64.8)	107 (34.0)	2 (0.6)	2 (0.6)		

Educational status	Illiterate	Below primary	Primary completed	Secondary	Higher secondary	Graduate and above
	45 (14.3)	56 (17.8)	140 (44.5)	39 (12.4)	20 (6.3)	15 (4.8)

Occupation	Unemployed	Home maker	Student	Unskilled labour	Skilled labour	Service/self-employed
	81 (25.7)	24 (7.6)	42 (13.3)	95 (30.2)	40 (12.7)	33 (10.5)

Socioeconomic status (Prasad scale)	Class I	Class II	Class III	Class IV	Class V	
	10 (3.2)	26 (8.3)	49 (15.5)	150 (47.6)	80 (25.4)	

Type of AED used	Conventional AED	Newer AED	Both			
	252 (80)	24 (7.6)	39 (12.4)			

**Table 2:** Distribution of the study population according to the average drug expenditure (*n* = 315).

Expenditure/ month (INR)*	Rural		Urban		Total	
	No.	%	No.	%	No.	%
Free of cost	53	67.9	166	70.0	219	69.5
1/— to Rs. 100/—	5	6.4	5	2.1	10	3.2
101/— to Rs. 200/—	2	2.6	5	2.1	7	2.2
201/— to Rs. 300/—	5	6.4	35	14.8	40	12.7
301/— to Rs. 400/—	5	6.4	11	4.6	16	5.1
401/— to Rs. 500/—	2	2.6	4	1.7	6	1.9
>500/—	6	7.7	11	4.6	17	5.4

Total	78	100	237	100	315	100

Mean ± SD (INR)	120.71 ± 219.93		95.93 ± 168.81		102.06 ± 182.74 *U* = 764.50, *P* = 0.304	

*INR: Indian rupees.

**Table 3:** Distribution of the study population according to the average travel expenditure for their visit to Medical College Neuromedicine OPD (*n* = 315).

Expenditure/ month (INR)*	Rural		Urban		Total	
	No.	%	No.	%	No.	%
Nil	0	0.0	24	10.1	24	7.6
1–25	0	0.0	87	36.7	87	27.6
26–50	34	43.6	104	43.9	138	43.8
51–75	22	28.2	16	6.8	38	12.1
76–100	18	23.1	4	1.7	22	7.0
>100	4	5.1	2	0.8	6	1.9

Total	78	100	237	100	315	100

Mean ± SD (INR )	66.47 ± 31.25		29.36 ± 18.36		38.55 ± 27.39 *U* = 1769.5, *P* = 0.001	

*INR: Indian rupees.

**Table 4:** Distribution of the study population according to the average expenditure for food and lodging for their visit to Medical College Hospital (*n* = 315).

Expenditure/ month (INR)*	Rural		Urban		Total	
	No.	%	No.	%	No.	%
Nil	2	2.6	104	43.9	106	33.7
1/— to Rs. 25/—	46	59.0	121	51.1	167	53.0
26/— to Rs. 50/—	26	33.3	12	5.1	38	12.1
51/— to Rs. 75/—	2	2.6	0	0.0	2	0.6
>75/—	2	2.6	0	0.0	2	0.6

Total	78	100	237	100	315	100

Mean ± SD (INR)	26.28 ± 14.04		8.97 ± 9.68		13.25 ± 13.22 *U* = 2515.0, *P* = 0.001	

*INR: Indian rupees.

**Table 5:** Distribution of the study population according to the average total expenditure for treatment of epilepsy (*n* = 315).

Expenditure/ month (INR)*	Rural		Urban		Total	
	No.	%	No.	%	No.	%
Nil	0	0.0	11	4.6	11	3.5
1–200/—	46	59.0	145	61.2	191	60.6
201–400	15	19.2	45	19.0	60	19.0
401–600	5	6.4	19	8.0	24	7.6
601–800	5	6.4	12	5.1	17	5.4
801–1000	2	2.6	1	0.4	3	1.0
>1000	5	6.4	4	1.7	9	2.9

Total	78	100	237	100	315	100

Mean ± SD (INR)	306.67 ± 340.59		191.28 ± 229.16		219.85 ± 265.34 *U* = 3575.50, *P* = 0.212	

*INR: Indian rupees.

**Table 6:** Distribution of the average total expenditure for treatment of epilepsy according to mode of therapy (*n* = 315).

Expenditure/ month (INR)*	Monotherapy		Polytherapy		Total	
	No.	%	No.	%	No.	%
<200	121	59.9%	81	40.1%	202	100.0%
201–400	38	63.3%	22	36.7%	60	100.0%
401–600	10	41.7%	14	58.3%	24	100.0%
601–800	5	29.4%	12	70.6%	17	100.0%
Above 800	0	00.0%	12	100.0%	12	100.0%

Total	174	55.2%	141	44.8%	315	100.0%

Mean ± SD (INR)	156.34 ± 162.89		298.23 ± 337.52		*U* = 9414.5, *P* < 0.001	

*INR: Indian rupees.

**Table 7:** Distribution of average total expenditure for treatment of epilepsy according to status of response (*n* = 315).

Expenditure /month (INR)*	Not responded		Responded		Total	
	No.	%	No.	%	No.	%
<200	8	4%	194	96%	202	100.0%
201–400	2	3.3%	58	96.7%	60	100.0%
401–600	1	4.2%	23	95.8%	24	100.0%
601–800	6	35.3%	11	64.7%	17	100.0%
Above 800	3	25%	9	75%	12	100.0%

Total	20	6.3%	295	93.7%	315	100.0%

Mean ± SD (INR)	461.25 ± 351.77		203.49 ± 250.93		*U* = 1450, *P* < 0.001	

*INR: Indian rupees.

**Table 8:** Distribution of the study population according to the presence of wage loss due to their visit to Medical College Hospital in the last three months (*n* = 315).

Wage loss	Urban	Rural	Total	*Z*	*P*
Present	95 (40.08%)	40 (51.28%)	135 (42.86%)	1.60	0.109
Absent	142 (59.92%)	38 (48.72%)	180 (57.14%)		

Total	237 (100%)	78 (100%)	315 (100%)		
